# The State of Music-Based Interventions for Mental Illness: Thought Leaders on Barriers, Opportunities, and the Value of Interdisciplinarity

**DOI:** 10.1007/s10597-021-00843-4

**Published:** 2021-06-08

**Authors:** Tasha L. Golden, Elliot Tetreault, Caitlin E. Ray, Maria Nagae Kuge, Alyssa Tiedemann, Susan Magsamen

**Affiliations:** 1 Johns Hopkins University, Baltimore, USA; 2 University At Albany, Albany, USA; 3 University of Louisville, Louisville, USA; 4 University of Florida, Gainesville, FL USA

**Keywords:** Interdisciplinary, Mental illness, Music, Mental health, Qualitative, Transdisciplinary

## Abstract

Hundreds of studies regarding music's effects on mental health have accumulated across multiple disciplines; however, access to and application of music as a support for mental health remains limited, due in part to the multidisciplinary nature of related research and difficulties synthesizing findings. This qualitative study is the first to address these barriers by gathering current thought leaders and stakeholders at intersections of music and mental health, representing multiple disciplines and backgrounds, to (1) document understandings of and recommendations for the field, and (2) examine how views converge or conflict. Participants (n = 36) viewed preliminary results of a global scoping review, then engaged in focus groups which were transcribed and de-identified for analysis. An interdisciplinary research team coded and iteratively analyzed transcripts. Six themes emerged: Barriers to Quality/Improved Research, Disciplinary Differences, Research Recommendations, Implementation and Access, Public Perception and Education, and Need for Training. Discussions offered wide-ranging observations and recommendations while revealing challenges and opportunities related to interdisciplinary work. Findings indicate broad agreement regarding current barriers and opportunities at intersections of music and mental health. While highlighting challenges, participants also indicated multiple avenues for advancing research quality, intervention effectiveness, and equitable access to music as a support for mental health. Responding to the study’s illumination of the benefits and challenges of interdisciplinary work, four brief recommendations are offered to support future efforts.

## Introduction

Since the mid-twentieth century, studies regarding music’s effects on mental health have steadily accumulated across multiple disciplines, with a substantial increase over the last 15 years (Golden et al. 2021). Hundreds of publications now point to a growing, multidisciplinary interest in the utilization and study of music as a support for mental health, emerging from fields such as psychology (McFerran et al. 2018; Pezzin et al. 2018), music therapy (Gold et al. 2006; Moe, 2002), neurology (Tan et al. 2016; Ventouras et al. 2015); nursing (Pölkki et al. 2012; McAffrey and Locsin 2002), and psychiatry (Grasser et al. 2019; Grocke et al. 2008), among many others. Study settings range from community-based programs for mood elevation or stress reduction to clinical interventions designed to treat serious mental illness (SMI). Results indicate that music can provide health benefits such as improved immune response and enhanced coping and emotional regulation (see Burrai et al., 2019; Pezzin et al., 2018; Rollins & King, 2015). They additionally suggest that the incorporation of music into mental health initiatives offer benefits associated with general arts engagement—such as increased social connectivity (Fancourt et al., 2016; Kreutz, 2014), improved health care delivery (Jersky et al., 2016; Nielsen et al., 2017; Pearl & Greenberg, 2020), and increased resilience (Gupta & Singh, 2020).

Despite accumulating studies and positive findings, access to and application of music as a support or treatment for mental health remains limited—as does information regarding the mechanisms by which music delivers benefits. These limitations can be traced in part to the interdisciplinary nature of research related to music and mental health, combined with the difficulty of communicating and coordinating findings across disciplinary boundaries (see Domino et al., 2007). For example, disciplines involved in music and mental health research vary not only in how they define terms, but also in their selected outcomes and outcome measures, and in their practices for conducting and reporting research (see Golden et al., 2021). This variety limits researchers’ and practitioners’ ability to synthesize evidence, generate shared best practices and clinical practice guidelines, and promote relevant policies.

These ongoing barriers to the advancement of mental health practice indicate the urgent value of bringing together thought leaders from relevant disciplines—along with individuals with lived experience with SMIs—for intensive dialogue regarding the state of the field of music and mental health. This qualitative study is grounded in such an initiative. To the authors’ knowledge, it is the first study to have convened diverse key stakeholders at intersections of music and mental health for the express purpose of identifying how they individually and collectively understand and describe the field, and to render explicit the ways in which their views may converge or conflict. The study also aimed to identify how participants perceived existing gaps and densities in the research landscape, and to gather their recommendations for next steps. Findings illuminate barriers and opportunities for the interdisciplinary work needed to improve effectiveness of and access to music-based supports for mental health.

## Methods

### Participants

This study was developed as a collaboration between One Mind and the International Arts + Mind Lab (IAM Lab) at Johns Hopkins University School of Medicine. The initial task of this collaboration was to conduct a comprehensive international scoping review of the literature regarding uses of music in the treatment of serious mental illness (SMI). Scoping reviews, as described by the Joanna Briggs Institute (2015), are intended “to map the key concepts underpinning a research area as well as to clarify working definitions, and/or the conceptual boundaries of a topic” (p. 6). They differ from systematic reviews in that they do not include quality assessments of included studies, and therefore do not generate meta-analyses or recommendations for practice. Instead, scoping reviews deliver a landscape of an emerging topic area—illuminating gaps and barriers in extant research and indicating key research opportunities moving forward. The scoping review for this initiative, titled “The Use of Music in the Treatment and Management of Serious Mental Illness: A Global Scoping Review of the Literature,” was published in *Frontiers in Psychology* (Golden et al., 2021).

The second task of the initiative, represented herein, was to convene a “think tank” to examine the scoping review’s preliminary findings and offer an interdisciplinary response. This think tank included thought leaders from psychology, neurology, psychiatry, music, media, music therapy, neurosurgery, public health, and policy who had expressed interest in—or conducted programs or studies related to—uses of music in the treatment of SMIs. It also included individuals with lived experience with SMIs, whose treatment experience(s) had incorporated music.

### Data Collection

Study participants were identified and recruited based on pre-existing relationships cultivated by One Mind and the IAM Lab. As recognized leaders in research regarding intersections of music and mental health, both organizations offered robust networks of prominent researchers, diverse program developers, clinicians and practitioners, and individuals with lived experience who regularly provide expertise regarding their experiences and treatments. One Mind sent invitations to 42 individuals, with 36 accepting.

Each participant attended a 60-min virtual presentation of preliminary results of the scoping review. Subsequently, participants were divided into six focus groups consisting of four to six members—each of which was curated by the IAM Lab to represent as many disciplines as possible. Each group engaged in a 60–90-min discussion facilitated by a member of the IAM Lab, using a formal facilitation guide to support consistency across conversations (Appendix B). Participants were asked about their initial responses to the content of the presentation; they were then asked to reflect upon any research gaps or densities that stood out to them based upon their histories or expertise, and to share their views regarding key opportunities to advance work at the intersection of music and SMIs.

These focus groups were followed by a final 90-min, full-group meeting attended by 27 participants. During this meeting, a representative from each focus group shared key points that had emerged in their conversations, with the goal of allowing the full group to integrate each group’s key results. After this, each participant was asked to briefly state what they believed would be the best next step to advance work at the intersection of music and mental health. All eight group meetings were recorded; as the initial call featured only a presentation regarding the scoping review, only the six focus group meetings and the final call were transcribed for analysis.

### Analysis

Results were analyzed using a theoretical thematic analysis consistent with steps described in Braun and Clarke (2006). A six-member research team undertook this process, including a lead researcher, coordinator, and four researchers from separate institutions—whose roles involved analysis and authorship. This team represented multiple institutions and disciplines including public health, arts in medicine, rhetoric, and psychology. Only the lead researcher had been present on any calls with study participants.

To begin analysis, one of the four researchers read all seven transcripts in advance of the full team and generated a preliminary codebook. Subsequently, the four researchers worked in pairs, with each pair being assigned three or four transcripts to read. Each individual read their assigned transcripts with the goal of identifying any codes not already captured in the codebook; they then met as pairs to review findings and reach consensus. Finally, each pair brought their findings to the full team to finalize codes for analysis. As needed, the lead researcher offered input and clarity from having attended calls with study participants. This process generated a codebook with 33 codes; each pair then utilized this codebook in the final coding process.

After coding, the research team assembled to discuss themes and subthemes in the transcripts. In light of the generated themes, codes were reconsidered and combined to generate a total of 26 unique codes (see Appendix A). A frequency count of codes was created, with coders selecting key examples from the transcripts to illustrate each identified code.

This study was reviewed and approved as exempt by Johns Hopkins Institute’s Internal Review Board. All group meetings were recorded with permission of members, and all members were de-identified during the transcription process, preceding analysis. This ensured anonymity of the participants and reduced risk of coder bias.

## Results

Six themes emerged from analysis: Barriers to quality/improved research, disciplinary differences, research recommendations, implementation and access, public perception and education, and need for training. Each theme is described in detail below.

### Barriers to Quality/Improved Research

As study participants discussed needs and opportunities to advance efforts at the intersection of music and mental health, they illuminated several barriers to quality research, coded as *Disciplinary Differences*, *Methodology and Measurement*, *Ethics / Duty to Patients*, and *Gaps in Data and Reporting*. The concept of Disciplinary Differences was so prominent in the data that it was explored as a separate theme, discussed in the next section.

#### Methodology and Measurement

Participants pointed to a “lack of rigor in the studies,” including difficulty determining what measures were used, a lack of intervention and outcome information, and a reliance on anecdotal reports, which one participant even described as “usually just quite frankly bullshit.” As discussed below, “rigor” itself was at times an ambiguous term in these conversations, tied to participants’ own disciplinary backgrounds, areas of expertise, and assumptions. Overall, participants described improved rigor as a means of generating better research to advance cross-disciplinary efforts to improve mental health outcomes through music.

Participants also discussed a lack of “mechanistic grounding” in the studies included in the scoping review.^1^ They suggested that interventions need to be mapped to “biological mechanisms and behavioral properties;” because, as one participant noted, “mechanism approaches ultimately give you the best therapies.” While many participants agreed with this assertion, others noted potential difficulties in identifying biological mechanisms within mental health research. For example, one participant argued that “There’s been no brain marker” for mental health conditions, “no body marker, approved or recognized yet, for any DSM diagnosis exam.” As a result, this individual asserted, measuring *symptoms* is critical to showing improvement in a given mental health condition—regardless of whether the mechanism by which that improvement occurs is well understood.

While discussing measurement, participants noted that certain aspects of music interventions may be difficult or impossible to measure right now, yet they nevertheless have positive effects on mental health. One participant asked, “How do we even begin to quantify and qualify the experience of music?”, while another contended that "music is a subjective experience; it’s hard to fully quantify what it does.” This was not offered as a reason *not* to study music's effects; however, participants emphasized a need to recognize existing limits. Notably, despite current complications in quantifying the “human experience” of music, participants acknowledged that it is nevertheless a “space that is very ripe for growth and development.”

#### Ethics / Duty to Patients

An additional noted barrier to quality research involved the ethics of treating individuals with mental health diagnoses. When discussing the creation of rigorous mechanistic studies as a basis for moving the field forward, a music therapist (MT) pointed out that MTs who use music-based interventions to treat patients “can't stop the treatment to do a study in order to be clinically rigorous.” MTs also argued that therapists are required to do what is best for each patient or client; as a result, they expressed concern about research-based intervention protocols that may preclude a therapist's ability to adapt, customize, and modify practices based on what they recognize as best for their patients. They therefore noted that mechanistic research may require separate trials, outside of MT clinical spaces. Finally, participants expressed ethical concerns related to research timetables, with one MT inquiring, “Can we wait 100 years until all the research is there?” Several participants argued that postponing music-based treatments until certain types of studies are completed could mean withholding opportunities for health benefits.

#### Gaps in Data and Reporting

Inadequate reporting practices also emerged as a significant research barrier, as they create difficulties in synthesizing evidence, replicating studies, and learning from previous efforts. Participants discussed the fact that many studies in the scoping review had not provided basic demographic information such as race or ethnicity and gender; many studies also failed to report specifics about the musical activities they entailed, and who facilitated them. Summarizing this, one participant stated, “[T]he lack of rigorous reporting…means you can't replicate [the studies] because you don't really know what they did."

Participants also expressed a desire to know more about how and why decisions are made to pursue music-based interventions for particular SMIs, noting a discrepancy “between how prevalent a certain severe mental illness is, versus how much it's being studied in this space.” Participants agreed that a future step should be to urge researchers to clarify how they arrived at decisions regarding which condition(s) to study, among which populations/groups, using what activities/interventions. In general, a lack of common reporting guidelines or expectations was recognized as a barrier for synthesizing evidence, improving practice, and aiding communication to the public. Additional reporting issues are documented under “Research Recommendations,” below.

### Disciplinary Differences

#### Language, Definitions

Discussions illuminated several barriers and tensions specific to working and communicating across disciplines, including disparate understandings of key terms and concepts. One participant noted, “We don’t have a unified taxonomy. We don't have a common language yet. Studies use a lot of different terms, and so it’s hard to actually get to what you’re looking for in the literature.” Participants also observed that the scoping review had revealed a lack of consensus regarding the term *music* itself: “In a lot of the studies,” a participant noted, “they use the term music but don’t define what the music is. And that’s always been the big challenge because music could be anything from vibration to rhythm, to harmony, to personally preferred songs.” In general, participants described a need to develop and iterate on shared definitions. They suggested that formal recommendations be made to generate a unified vocabulary: “We need recommendations for key terms, common taxonomy, core outcomes, recommendations to say if you’re studying in this area, try to include this type of measure, or here’s the low hanging fruit for where we have opportunities to find strong outcomes.”

Basic concepts were also said to require further clarity. A participant argued that “the biggest misperception is that *all* music engagement is somehow therapeutic in the strict sense of what therapy and medicine is," and that there needs to be a clearer distinction “between ‘music and health' and 'music therapy.” In other words, while music interventions can be therapeutic, they do not all qualify as “music therapy.” They noted that “there’s a difference between going to the gym and working with a trainer when you're healthy or trying to get healthier, as opposed to working with a physical therapist when you have some type of disease or disorder.”

#### “Rigor” across Disciplines

Rigor emerged during analysis as a particularly provocative concept for interdisciplinary research and practice. One participant stated that “it is very hard…to bring the arts in as an intervention," and participants traced this difficulty to broad tensions between the arts and sciences—including differing understandings of and assumptions regarding rigor and quality. For example, while discussing a lack of methodological rigor in the scoping review’s studies, a participant asked if a lack of rigor in arts research is typical of the field. Another responded that research issues were due not to a general lack of rigor in the arts, but to the need to optimize and integrate the strengths and knowledges of relevant disciplines:

“I really think that in order for this field to move forward, we really have to encourage really interdisciplinary teams to work together. So you know, for any studies to actually have the level of rigor that [other participant] is talking about, …we really need to encourage partnership between scientists, music professionals, clinical trialists, people that develop behavioral interventions, and even technology development folks because we want to have a better delivery system for the intervention. So to me it’s essential that we really promote the formation and the utilization of those interdisciplinary teams.”

Here, “rigor” is constructed not as a property of one field of study or as something that a given field is missing, but instead as the outcome of strong interdisciplinary collaborations. For example, one participant observed, “In almost every one of the studies that address this topic…, there wasn't a recognizable music neuroscience person involved. They were people who do other things and decided to get involved with music. And I think that's where a lot of errors creep in, when you think that you're an expert on something…" In general, participants emphasized that rigorous studies emerge from robust partnerships—enriching the field overall.

#### Tensions across Disciplines

Analysis indicated additional tensions across disciplines and sectors. For example, when discussing how to “engage pharma,” one participant suggested researchers focus on framing music interventions as a supplement to pharmaceutical solutions, so that “people wouldn't feel threatened that we are actually advocating one thing instead of more an integration.” Another participant indicated “that psychiatrists in particular are very resistant to any other [non-pharmacological] approach to treatment,” and that “someone versed in marketing or communication skills” may need to “shift that paradigm a little bit…otherwise, it’s going to be hard to scale up even though we might have good data.” They later added, “If the physicians are not part of the solution, it might not go anywhere.” These discussions indicated that power differentials among sectors and disciplines may pre- or over-determine research and practice.

#### Structures to Encourage Interdisciplinary Work

Despite clear agreement that interdisciplinary research needs to improve, participants argued that productive interdisciplinary collaborations—including shared definitions and research practices—will not simply happen on their own. Instead, structures must be put in place to encourage and incentivize interdisciplinarity. A lack of such structures was seen as actively precluding beneficial collaborations; recommended changes included encouraging funders and initiatives to *require* research teams to be interdisciplinary—for example, by including music therapists or trained musicians, neurologists, psychologists, and individuals with lived experience. Referencing a recent national health funding initiative for which research teams were required to be interdisciplinary, one participant noted that the strength of the resulting applications actually persuaded leaders of the initiative’s value—which they had previously considered risky and unconventional.

In addition to lacking structures to support interdisciplinarity, there are often barriers to their implementation. For example, one participant stated that their organization had wanted to offer “a matchmaking type of thing” on their institutional website—providing a “list of investigators” and practitioners so that parties could search and generate collaborations. However, the participant lamented that they “can't do that legally.” Notably, another participant saw this as a case in which arts organizations such as the National Endowment for the Arts (NEA) could provide structural assistance to “really kind of galvanize the field and potentially make those connections.”

Participants also observed that creating structures that support interdisciplinary research could become part of the mission of the immediate gathering of which these focus groups were a part. For example, the think tank could recommend "that interdisciplinarity be a loud emphasis [in music and SMI research], that always includes a clinician—a music therapist—who has questions that can be raised from their own clinical experience, but then also includes people who have perhaps had more rigorous research training and experience." They posited that the think tank's ability to make and share recommendations, while also supporting interdisciplinary connections, could help build stronger research teams—and thus a stronger field overall.

### Research Recommendations

The third theme was the most prominent; a significant portion of focus group conversations were devoted to recommendations for helping improve research at intersections of music and mental health.

#### Reporting Practices

First mentioned under “Barriers…,” concerns about reporting practices also framed recommendations. For example, a participant noted that “some studies didn’t seem to include like, basic information that you would need, like the gender or ethnicity of the people that were studied;” another participant stated simply, “You can't even figure out what the actual intervention was.” This lack of detailed reporting was described as “really surprising,” with another participant noting that "the lack of rigorous reporting—which means you can’t replicate them because you don't really know what they did—was quite shocking.”

#### Gaps in the Research

Study participants extensively discussed current gaps in the research that they want to see addressed. These have been documented, along with reporting recommendations, in Table 1.

**Table 1:** Research recommendations

Reporting recommendation	Purpose/Value
Develop “reporting guidelines specific to music interventions”	Address the unique importance in music-based interventions of details such as setting, who facilitates, how the intervention was developed, and whether it is offered in conjunction with or in place of other treatments
Improve reporting of details (genre, tempo, facilitator, dose/duration, whether the activity was group or individual, who chose the music/genre, what other types of treatment participants had already tried, participant demographics)	Better understand how and to what extent these variables determine or contribute to outcomes, and for whom

Research Recommendation	Purpose/Value
Study how variables such as specific diagnoses, activity variables (such as genre or tempo of music), and experience or history with music affect outcomes for various conditions	If music is found to have “reliable and consistent” effects given specific variables, then researchers and clinicians could develop “a particular methodology that will apply to anybody who has a [given] diagnosis”
Examine potential for developing “a screening test for music,” that could identify which types of music are likely to be most conducive, and which types may be likely to cause adverse reactions	This would allow selection of music based on the current patient’s condition, preferences, and needs, potentially rendering music-based interventions safer, more effective, and more immediately helpful. One participant compared this idea to an “allergy test for music.”
Investigate what patients or participants are experiencing (emotionally, psychologically, etc.) while listening to or engaging with music	Generate increasingly granular understandings of how and why listeners are affected by music, including which aspects or moments of music tend to generate beneficial responses, and for whom
Investigate whether “there are certain musical activities that strengthen or complement” biological or psychological factors that have proven links to causes or symptoms of mental health conditions	“[I]f we could start to make connections between…somebody who has a certain illness [who] might benefit from extending, you know, short term memory, extending executive function…and [if] we could see that a particular musical activity would in fact help with that…That's the kind of a chain I could imagine.”
Study how facilitators affect interventions	Determine extent to which training, history, approach, relationship to patients, demographics, and other facilitator details affect outcomes of music-based interventions
Design a longitudinal randomized controlled trial (RCT) comparing outcomes for similar individuals who undergo music therapy versus no/delayed treatment	Additional insight regarding efficacy of music-based interventions

#### Consolidating and Reviewing Extant Research

Contrasting with recommendations to initiate new studies, many participants prioritized further consolidation and examination of extant evidence. One individual suggested “curat[ing] a critical summary of like 10 to 20 of the most interesting articles” from the scoping review, while another recommended gathering “the 10 best examples of a particular type of study” as exemplars. (Participants did not suggest criteria by which curated or exemplar studies should be selected.) Another participant argued that, “Until we evaluate what the available data tell us, it seems premature to proceed with any new studies.” Still others noted that a greater ability to combine information from studies across disciplines—a capacity that relies upon improved interdisciplinary collaboration—could generate "best practices" for both research and practice at intersections of music and mental health.

#### Design Research for Implementation

Lastly, in keeping with an overarching focus on implementation, participants noted that research must be able to be put into practice. They therefore recommended that implementation be considered from the earliest stages of study design, so that research can be “effective and efficient in getting to what could be interventions.” Echoing the value of interdisciplinary research, participants suggested that researchers consult with music therapists “to inform the[ir] questions…Because sometimes the actual questions that the researchers are asking are, you know—it would be really difficult to translate them either directly or four steps down the road into something that's clinically useful.”

Participants also asserted that research should be designed to measure multiple outcomes, so that varied practices could be implemented at corresponding levels of evidence. For example, one individual argued that the variable success demonstrated by standard mental health treatments indicates that *any* value added by music-based approaches is worth pursuing: “[F]or some people who *don’t* respond to traditional therapy, which is a large number, you might add music and [generate] a better outcome mixed with traditional stuff.” Another asserted that if evidence exists “that [music] can improve some of the symptoms of one of these illnesses,” then even if we do not yet know whether a given music-based intervention can, for example, “cure or significantly ameliorate major depression,” one could still state that it offers “a way to enhance resilience or other human performance.” Availability of these practical applications requires that studies examine music as an adjunctive or additive therapy, and that study designs include qualitative measures related to symptoms, function, and quality of life.

### Implementation and Access

The fourth theme emerging from the data involved the question of how music-based interventions can be implemented and accessed. Participants’ major concerns included insurance coverage, expanded access, and necessary policy changes.

#### Health Insurance Coverage

The cost of healthcare was such a primary concern that all conversations about how to implement music-based interventions turned into conversations about how to get health insurance to cover it. One participant put it in stark terms: “I’ve been in so many of these strategic meetings to try to develop the evidence of a particular intervention and then try to move it to implementation. By implementation, if it’s in the medical arena, that means getting a billing code.”

Other participants observed that, even when music-based therapies have a billing code, covering the costs of treatment remains difficult under reimbursement models.^2^ “[Y]ou sort of have to petition for any kind of Medicare reimbursement or private insurance…there's really no easy mechanism for people to use their insurance to pay for it.” As a further barrier to coverage, participants noted that even the particular delivery model for music-based interventions (such as primary care, medical home, or telemedicine) can affect insurance approval.

Conversations also noted the relative lack of adverse side effects when using music in the treatment of mental illness, seeing this as an argument for increased coverage. According to one participant,

Study after study ha[d] failed to find side effects to this intervention. So, in that sense, if music were to be compared directly to a more established reimbursed intervention, say…for depression or schizophrenia, found to be equivalent, but without the side effects, that clearly is a win logically. I would think that would be an argument for reimbursement. So, that's maybe something that we could emphasize more.

#### Policy Change

Participants also discussed how policy and health care practice could together advance the use of music-based interventions and increase recognition of music engagement as potentially preventative. For instance, participants mentioned that the U.K. and locations in the U.S. have trialed “social prescribing”—a practice in which health care professionals refer patients to non-clinical services such as arts-based activities, volunteering, gardening, or sports.^3^ “Massachusetts is using music right now for social prescribing for stress and anxiety;” one participant observed; “it’s the first time it's been done in the United States.” However, in keeping with a focus on *costs* of treatment, this participant was concerned that social prescribing is still dependent on insurance coverage, adding that “there’s a payer model issue with social prescribing that I think has to be addressed.” In short, the ability to prescribe music-based activities for health conditions would require changes to health policy to accommodate coverage.

#### Expanded Delivery/Access

In addition to insurance coverage, participants offered other ideas for expanding access to music-based interventions—such as utilizing telemedicine to provide remote access to MTs. Noting that delivery of health care has shifted during the COVID-19 pandemic, one participant stated, “Maybe what we can show is that it’s entirely possible to have effective music interventions that are remote…and maybe that’s something that really should take strong consideration to solve not only the problem of access in places where…you can't afford to have a music therapist on staff, or can't afford to have a musician coming in.”

### Public Perception and Education

For the fifth theme, participants argued that public perception and education regarding music-based interventions were integral for obtaining additional funding for related research, and for developing more support among stakeholders—including health care providers and individuals with lived experience: “[Y]ou have to persuade people that this works, and this is worth the money.” Participants also contended that public perception is needed to develop advocacy for relevant policymaking, including movement toward social prescribing. One individual argued that changes to policy, insurance coverage, and health care practices may not happen "until people demand it because they’re reading more about it…. I think building an informed and engaged…public [could] drive…more research.” Other participants agreed, with one asserting that insurance coverage could be improved via increased public pressure on insurance companies and processes. An additional recommendation was to persuade professional organizations to draft bipartisan letters to Congress requesting that music therapy be added to a list of reimbursable services. Such a letter would mean that “insurance companies could then consider” the request. Another participant argued that “a change in public perception” is “what drives funding” and policy changes; they hypothesized that if “enough people lobby their Congresspeople,” such pressure could generate partial or state by state advances—eventually leading to full coverage.

It is important to note that, any time that participants emphasized public awareness, other participants expressed concern about focusing on public education campaigns before we have more (and better) research. One participant asked: “What do you tell the public when you don’t have a firm basis on what’s the right thing to say?” Another agreed: “the science needs to come first.” More bluntly, a participant argued that it would be “nonsense” to “put out little, you know, pretty web brochures and stuff saying, ‘Oh, this is so valuable,’” before having the "medical science, lab science” to “tell you this is true.”

### Need for Training

The sixth and final theme involved the need for new or improved training and education processes for music therapists (MTs), intervention facilitators (who are not MTs), and researchers. Regarding MTs, participants identified a need for increased opportunities to become a music therapist—due to a concern that the current number of MTs will not be able to meet rising demand. One individual indicated that "we need to have something more like 200,000 music therapists out there to meet the needs of the country, which means we need…more programs that have, you know, at least a capacity of 150 students.”

Participants also asserted that providing MTs with more rigorous training in conducting research would allow them to contribute more knowledge across fields and disciplines. While noting that interdisciplinary teams of both MTs and full-time researchers could help bridge gaps in MT research training, participants expressed a specific need for MTs to themselves have more research training. Although MT programs provide some such training, participants asserted that expectations of music therapy professionals and journals regarding rigor, reporting practices, and study designs appear to differ from expectations in fields such as psychology, neuroscience, and medicine. In response, participants argued that improving MT research training could help ensure that findings from music therapy studies are more readily synthesized with those of other fields—and that best practices are recognized and translated across disciplines. Unfortunately, structural and disciplinary constraints appear to limit MTs’ access to training. For example, a participant mentioned that it was difficult to find MTs who could participate in programs like the NIH Center for Translational Science (CTSA)—which invites individuals with PhDs to receive further training in clinical research methodology—because “we don’t have enough schools that offer doctorate level in…music therapy.”

Finally, participants observed a need to improve the training available for *any* facilitators of music-based health programs—whether MTs or not. Many of the studies examined in the scoping review had not specified *who* facilitated the music-based interventions, and many other studies indicated that the music-based aspect of their study had been facilitated by researchers and/or therapists with no reported training in music or the arts. This raised concerns among study participants, who emphasized the need to offer and standardize training for non-therapists. “I just reviewed a paper for *PNAS*,^4^” one participant relayed,

It was about music. And none of the people involved had ever done a music study. None of them were cognitive psychologists or perceptual psychologists. They didn't even know what kinds of things had to be balanced and accounted for. So, I wonder if because music is ubiquitous and everybody loves it, they think, Oh, well, I'll just jump in. But you know, I have a heart, but it doesn't mean I'm going to be pontificating about cardiology.”

## Discussion

This study generated considerable findings related to research barriers, disciplinary differences, implementation needs, and recommendations for the field. Participants generally agreed that music can and should be further explored and better understood via research, while acknowledging that many aspects of music are currently challenging to measure. Disciplinary differences in what constitutes rigor or conclusivity indicate potential barriers to agreement on what researchers or studies should be seeking, and how success will be defined. Conversations additionally revealed a tension between the value of offering music-based practices that SMI patients find to be beneficial—even when the mechanisms behind the benefits are not wholly understood—and the value of developing interventions and practices derived from robust understandings of the mechanisms by which music benefits SMI patients. Cost and health insurance coverage emerged as significant barriers to implementation and access.

Across focus groups, differing knowledge bases also suggested potential barriers. For example, MTs’ concern that rigorous research would preclude music therapy’s adaptability and customization overlooks complex intervention study protocols—which allow for modifications. Similarly, researchers’ assumptions that a current lack of mechanistic studies means practitioners are not yet able to select music based on specific patient needs overlooks the considerable knowledge accumulated via clinical practice and training. Such oversights affirm that greater interdisciplinarity could increase the field’s capacity to respond to various practitioner, patient, and researcher challenges with appropriate, responsive study designs.

The bulk of dialogue was devoted to improving research and research reporting at intersections of music and SMIs. Despite participants' extensive familiarity with the field of music and mental health, many indicated alarm regarding inconsistencies and inadequacies in reporting practices. Responding to preliminary findings from a scoping review, participants made recommendations that could advance the field—including mechanistic studies, improved reporting of study details and participant demographics, longitudinal studies, and the provision of rationales for choice of facilitators, music-based activities, and genres. While some participants encouraged additional secondary studies, others prioritized new studies with specific protocols, outcomes, and standardized reporting practices as a baseline for future research.

In general, this study found that focus groups contended both with obvious disciplinary differences, such as in terminology used, as well as implicit differences regarding values and assumptions. For example, across focus groups, the notion of “being an expert” was not explicated—though it was likely understood differently by differing participants. Participants also did not discuss how the value placed on particular forms of “expertise” may result in the devaluation of other forms, and how differing valuations can affect research priorities, practices, and outcomes. Similarly, although terms like “rigor” and “success” were not clearly defined, various usages (“not an expert,” “lack of rigor,” “won’t succeed”) implied underlying assumptions that were not rendered explicit. As a result, participants could not ensure that terms and expressed values were shared and useful for collaboration. While challenges such as these are by no means particular to the group or topic at hand, this study affirms that, particularly when working across disciplines, tendencies to take one’s values or definitions for granted may preclude the explicit discussions and constructive arguments needed to establish robust common ground.

Public education presented another topic about which disciplinary assumptions or practices appeared to create barriers. Participants who advocated against focusing on public education—at least before more research is generated—were primarily in research-focused positions, while participants who prioritized public education were more likely to identify as MTs/practitioners, patients, or musicians. The former appeared to perceive the latter as being willing to launch campaigns about music's benefits with or without evidence; they also appeared to view research and public education as distinct, linear endeavors. By contrast, the latter group viewed public education as bound up with research, discussing it as both a precursor to research (by advancing demand and funding) and as an indication of success—with research ideally leading to implementation, improved outcomes, and widespread awareness. Explicit discussions of terms and assumptions may have aided participants in reaching common understanding on this topic.

Finally, participants noted the need for training to (1) increase the number of MTs; (2) improve MT research skills; and (3) help ensure that anyone facilitating a music-based activity as part of a health intervention has baseline skills, in order to minimize potential harms while optimizing benefits. Regarding non-MT facilitators of interventions, participants theorized that familiarity with music may lead researchers to believe they can capably facilitate music-based experiences, regardless of their level of training in music, psychology, therapies, or the particular health condition(s) being addressed by music-based interventions.

## Recommendations

The current study indicated that optimizing music’s benefits for mental health demands multiple sets of expertise, including in music, music facilitation, research (in multiple disciplines), and public dissemination. In general, solving intractable problems related to health and wellbeing demands innovative, interdisciplinary approaches capable of generating, studying, and iterating complex interventions (Golden & Wendel, 2020; Mitchell et al., 2015; Sonke et al., 2019). Such innovation is aided by cross-disciplinary and cross-cultural research, which can improve creativity, problem-solving, dissemination, and potentially even citation rates (Yegros-Yegros, Rafols, & D’Este, 2015)—while also helping ensure that projects benefit from (and do not unnecessarily repeat) parallel research from other fields. Having detailed barriers to interdisciplinary research and practice illuminated by this study, the authors now identify four brief recommendations for improving interdisciplinary efforts.

### Prepare for Potential Challenges

It is important to be clear-eyed about the range of potential difficulties posed by interdisciplinary work, from structural barriers (see below) to challenges generated by definitional and procedural differences, unexamined assumptions, and power imbalances. With awareness, teams can take steps to ensure inclusive leadership, obtain guidance and support for cross-disciplinary and cross-sector communications, and craft collective group expectations regarding goals, processes, and shared spaces (see Lindgreen et al., 2020; Morss et al., 2018; Magsamen & Lohnes, 2017). More generally, explicit acknowledgement of common difficulties in interdisciplinary work helps convey that such problems are not a sign of failure; rather, they can be anticipated, accommodated, and overcome.

### Advocate for Structural Changes and Incentives

As participants noted, funders and institutions should be encouraged to require interdisciplinary research teams (see also Lindgreen et al., 2020; Yegros-Yegros, Rafols, & D’Este, 2015); similarly, publishing standards could be revised to explicitly value interdisciplinary studies. In addition, universities can be asked to provide quality spaces or programs explicitly designed to bring disciplines together, such as the Innovation and Entrepreneurship Institute at Temple University, the Cooperative Consortium for Transdisciplinary Social Justice Research at the University of Louisville, or the Ignite Research Colloquium at Oregon State University. Reward structures for university faculty could also be altered to incentivize interdisciplinary research. For more on top-down changes, see Morss et al., 2018.

### Choose a Framework

Interdisciplinary initiatives benefit from agreeing upon a shared research framework, which can help concretize processes and align goals. For example, the IAM Lab generated the Impact Thinking framework for interdisciplinary research, specifically designed for studies regarding intersections of the arts with health and well-being (Magsamen & Lohnes, 2017). The framework guides interdisciplinary teams through eight stages of the research process, including problem identification, study design, evaluation, and dissemination. Taking a more general approach to an interdisciplinary work, Allen Repko (2008) offered a 10-step process that was later reiterated by Szostak (2012). Many research frameworks are available; what matters is that the framework fit the project at hand, and that its usage is determined by collective (interdisciplinary) agreement.

### Consider Involving Specially Trained Facilitators

In the current study, focus group facilitation was supported by a common facilitation document, pre-meetings among facilitators to communicate goals and answer questions, and an emphasis on equitable inclusion of all participants. Based on findings, future initiatives could extend these benefits by ensuring that group facilitators also help participants: define terms, reach agreement on how various concepts are prioritized or valued, and converse explicitly about how individual backgrounds or disciplines may influence perceptions.

While the ability to offer such assistance may come naturally to some, the requisite sensitivity, patience, and listening and communication skills often stem from training and experience. As a result, interdisciplinary initiatives may wish to identify an individual or individuals trained in group facilitation, critical pedagogy, andragogy, or mediation to help guide and frame initial discussions among participants. This is not to suggest that conflict is inevitable, nor that project participants are incapable of high-quality communications on their own. Rather, it recognizes that interdisciplinary challenges need not be borne without support, and that the expertise of trained facilitators may free group members to focus on the central questions and problems of their project.

## Limitations

Robust coding and analysis in this study were supported by the interdisciplinarity of the research team. While it is theoretically possible that this interdisciplinarity rendered a focus on interdisciplinary challenges, it is more likely that resultant discussions regarding unshared definitions and perceptions, combined with multiply iterative processes, generated trustworthy insights to support an inherently interdisciplinary field.

A potential limitation lay in the fact that, although the value of inclusive dialogue was emphasized throughout, participants who identified as researchers appeared to hold more conversational power than did music practitioners or individuals with lived experience. Given that all discussions emphasized research practices, this weighting is not unexpected. However, interdisciplinarity is valued in part because it supports the re-viewing and re-cognizing of practices and opportunities from alternate and outside perspectives. Opportunities for insight and improvement may be missed if discussions adhere to assumed hierarchies of knowledge. Future interdisciplinary research and dialogue could therefore be enhanced by explicit acknowledgments of potential power dynamics, and by facilitators’ efforts to ensure all experiences and disciplines are equally represented. In addition, as suggested above, interdisciplinary collaborations will generate even greater clarity and productivity when they establish explicit, shared understandings of the underlying values and definitions that shape conversations and research.

## Conclusion

This qualitative study is the first to gather current leaders at intersections of music and mental health, representing multiple disciplines and backgrounds, in order to document their understandings of and recommendations for the field while also examining how their views converge or conflict. It presented a summary of themes that illuminate field-wide barriers such as a lack of standardization, the need for additional training, and disciplines’ differing terminology, values, and priorities. These findings inform the groundwork for future research and practice related to music-based mental health interventions. They also indicate that increased awareness and deliberation across disciplines will support future collaborators in addressing, discussing, and confronting roadblocks as they create and sustain partnerships and initiatives.

Conversations highlighted thought leaders’ significant level of agreement regarding the promise of music as a support for mental health, current research needs, implementation issues, and next steps. In particular, participants offered multiple recommendations to further the field, including structural supports for more interdisciplinary research, public education campaigns, additional secondary research, and the creation of research guidelines, among many others. Despite important gaps and barriers, it is apparent that multiple pathways exist to advance research quality, intervention effectiveness, and equitable access to music as a support for mental health, and that leaders across multiple involved disciplines see value in advancing this critical work.

## Footnotes

1 Notably, the inclusion criteria for the scoping review—the results of which focus groups were responding to— required that a given study examine music as a *treatment* or *support* for mental illness. In other words, mechanistic studies, such as those studying music's effects on the brain, may have been excluded if they were not additionally designed to treat or mitigate symptoms related to a serious mental illness.

2 “Healthcare reimbursement is defined as the process by which private health insurers or government agencies pay for healthcare providers’ services” (UIC, 2020).

3 “Social prescribing, also sometimes known as community referral, is a means of enabling health professionals to refer people to a range of local, non-clinical services. The referrals generally, but not exclusively, come from professionals working in primary care settings... Examples [of prescribed activities] include volunteering, arts activities, group learning, gardening, befriending, cookery, healthy eating advice and a range of sports” (Buck & Ewbank, 2020).

4 *Proceedings of the National Academy of Sciences.*
